# Kissing Balloon-Stent Technique for Simple Bifurcation Lesions

**DOI:** 10.3390/jcm13092645

**Published:** 2024-04-30

**Authors:** Adnan Kassier, Kameel Kassab, Tim A. Fischell

**Affiliations:** 1 Division of Cardiology, Mercy Heart Hospital, Springfield, MO 65804, USA; adnan.kassier@gmail.com; 2 Division of Cardiology, University of Missouri, Columbia, MO 65212, USA; 3 Division of Cardiology, Yuma Regional Medical Center, Yuma, AZ 85364, USA; 4 Division of Cardiology, Ascension Borgess Hospital, Kalamazoo, MI 49048, USA; tafisc@gmail.com; 5 Homer Stryker M.D. School of Medicine, Western Michigan University, Kalamazoo, MI 49008, USA

**Keywords:** balloon stent, bifurcation, coronary artery disease

## Abstract

**Background:** Coronary bifurcation lesions are commonly encountered during coronary angiography. The management of bifurcation lesions remains challenging, with various bifurcation techniques being available and outcomes varying depending on the Medina classification and operator experience. **Methods:** We present a short case series and the outcomes of a new bifurcation technique for the management of simple Medina ‘0,0,1’ and ‘0,0,1’ bifurcation lesions using the kissing balloon-stent technique (kissing BS). **Results:** We retrospectively identified 8 patients who underwent bifurcation stenting using the kissing Balloon-Stent technique, along with their clinical and angiographic follow-up outcomes. We also describe the benefits and limitations of the technique, delineate the potential mechanisms of target lesion failure, and outline appropriate patient selection. **Conclusions:** Kissing Balloon-Stent technique is a simple single stent technique that is safe and feasible in select patients with Medina ‘0,0,1’ and ‘0,0,1’ bifurcation lesions.

## 1. Introduction

Coronary bifurcation lesions represent up to 20% of all coronary stenosis [1]. These lesions are challenging and are associated with a higher rate of restenosis and repeat revascularization after percutaneous intervention [2]. There are several ways to manage these lesions, including using a single-stent technique (provisional stenting (PS)) or a two-stent strategy (the classic double kissing DK crush, mini-crush, nano-crush, culotte, T stenting, etc.) depending on the patient’s anatomy, as well as on the operator’s preference and experience. Multiple randomized trials have largely failed to show the superiority of one technique over another, except when treating left main bifurcation lesions. Although PS is preferred by most operators for most bifurcation lesions, depending on the anatomy involved, recent data suggest potentially better results with the DK crush technique in complex bifurcation cases, including left main bifurcation lesions [2,3].

Medina classification is the most widely utilized method to describe coronary bifurcation lesions in the literature. The bifurcation academic research consortium defines true bifurcation lesions by the presence of stenosis ≥50% diameter in the main vessel and the side branch. Under this classification, true bifurcations are considered to be either Medina 1,1,1, 1,0,1, or 0,1,1. All the rest of the bifurcation lesions are considered non-true bifurcations [4]. Medina classification helps clinicians to understand lesion distribution, plan percutaneous coronary interventions (PCI), and predict patient prognosis. According to the definition criteria [5], most bifurcation lesions are simple (e.g., a Medina classification of (0,1,0) or (0,0,1)) with the PS and one-stent technique currently being the most preferred method to treat such lesions. Registry data suggest that among all the bifurcation lesions, Medina 0,0,1 is the least prevalent at 4.1%, while Medina 0,1,0 is more prevalent at 14.9% [6]. In a large multicenter registry evaluating the outcomes of PCI treatment for bifurcation lesions, Medina 0,0,1 lesions involving the side branch ostium were associated with the worst clinical outcomes of one year [7]. In this registry, the rates of target lesion failure (TLF) and target vessel failure (TLV) for Medina 0,0,1 lesions were as high as 8.1% and 8.8% at the one-year point. For Medina 0,1,0 lesions, event rates were better, with rates of TLF and TVF at 3.4% and 3.8%, respectively [7]. This may be related to the morphology of these lesions being highly fibrotic or calcific, with a high risk of recoil and re-stenosis. 

There is limited data on managing these lesions, highlighting the need for better optimization of PCI results. In this paper, we introduce and describe a new stenting technique intended for the treatment of Medina 0,1,0 and 0,0,1 bifurcation lesions, named the kissing balloon-stent (kissing BS) technique. This paper presents a small case series that illustrates the safety, efficacy, and durability of results using this single-stent technique. We report eight cases performed at our center, along with long-term clinical and angiographic follow-ups. 

## 2. Methods

We implemented a retrospective cohort study design. All patients who ever underwent bifurcation stenting at Ascension Borgess Hospital using the kissing-BS technique were identified. We then conducted a retrospective chart review to identify both patient and procedural characteristics. Procedural details were gathered from the procedure notes, reports, and reviews of angiographic images and data. We collected data on any immediate post-procedural events and included the longest relevant follow-up data for all the identified patients. All the operators performing the procedure were experienced operators who performed bifurcation stenting regularly.

## 3. Technique Description and Rationale

**Figure 1:**
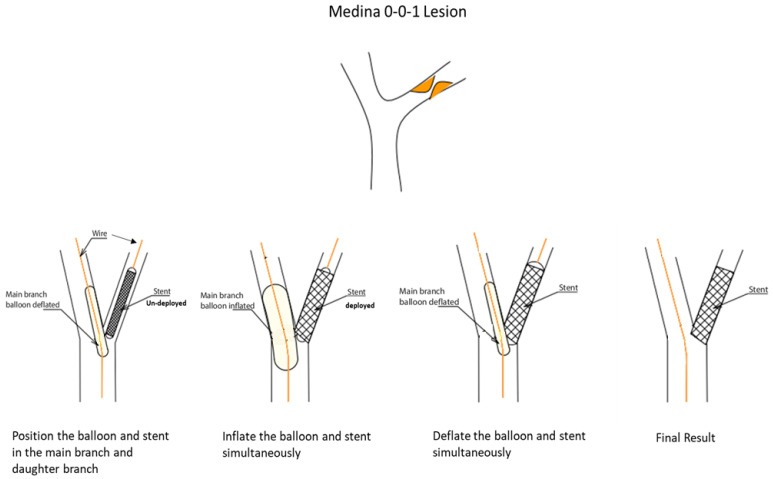
Step-by-step procedural description of the kissing balloon-stent technique.

The kissing BS technique can be utilized to treat simple bifurcation lesions, namely, Medina class (0,1,0) or (0,0,1), such as left anterior descending (LAD)/diagonal or left circumflex (LCX)/obtuse marginal (OM) bifurcations, where the obstructive disease involves only one branch of the bifurcation. The rationale is to protect the non-obstructed branch from carina and plaque shift during single-stent deployment. This plaque shift issue is not uncommon with the PS technique; it is widely described in the literature and has also been noted in everyday practice [8]. Plaque shifts usually occur when the disease involves the origin point of the branch, especially if the branch is positioned at <60°. With the PS technique, the operator either chooses to stent across the healthy branch of the bifurcation to cover the diseased segment or attempts to land the stent accurately at the origin of the branch without stenting across it. In both cases, plaque shift can occur, and operators usually opt to protect the healthy branch with a wire, should they need to salvage it (usually via stenting). A geographic miss is also another risk that can happen since the operator tends to position the stent slightly away from the bifurcation to prevent plaque shift, or, in other cases, protrudes too much of the stent into the non-stented vessel. Hence, we have developed the kissing BS technique to minimize plaque shift, help protect the healthy branch, and create a “nano-crush” of any small proximal stent element that may protrude into the non-obstructed vessel (Figure 1). 

A kissing-BS procedure can be performed using 6 French guide catheters. After wiring both branches of the bifurcation, one might first pre-dilate the diseased vessel. After pre-treatment, the operator delivers the stent across the target lesion, along with a short (15 mm) balloon, sized 0.9:1 (preferably a non-compliant balloon), into the non-obstructed branch they intend to protect. Then, the stent is positioned precisely, covering the lesion, and with minimal protrusion in the non-diseased vessel to avoid a geographic miss at the ostium. After inflation of the stent balloon and the verification of adequate positioning, the operator inflates the balloon in the side branch (typically 12–14 atm.), achieving a nano-crush “kissing BS”. Thereafter, the operator can increase or decrease the pressure either in the balloon or on the stent as they deem necessary to achieve optimal results. After a minimum of 30 s, the two balloons are deflated simultaneously to avoid any preferential plaque or stent shifting (Figure 1).

## 4. Results

Retrospectively, we identified eight patients with Medina 0,1,0 and 0,0,1 lesions who underwent bifurcation stenting at Ascension Borgess Hospital using the kissing-BS technique. The duration of follow-up, including both angiographic and clinical assessments, was variable, depending on the potential recurrence of symptoms, the need for repeat angiography, and the longest clinical follow-up duration available. Table 1 and Table 2 summarize the lesion characteristics and lesion outcomes. Below is a summary of the included cases.

## 5. Case Series

Case # 1

**Figure 2:**
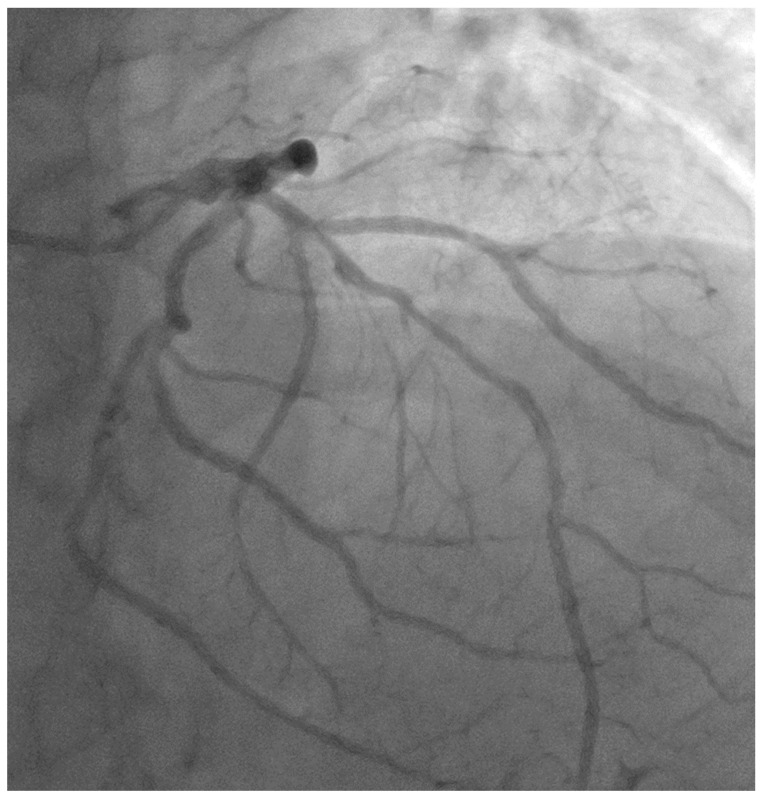
Image showing native mid-LAD disease starting at the diagonal bifurcation (Medina 0,1,0).

A 56-year-old gentleman with multiple risk factors, including hypertension, type II diabetes mellitus, peripheral arterial disease, and an active smoker, was referred for percutaneous coronary intervention (PCI) regarding a mid-LAD lesion starting at the diagonal bifurcation (Medina 0,1,0), after presenting with class III angina and recording a positive stress test for anterior ischemia (Figure 2, Video S1).

**Figure 3:**
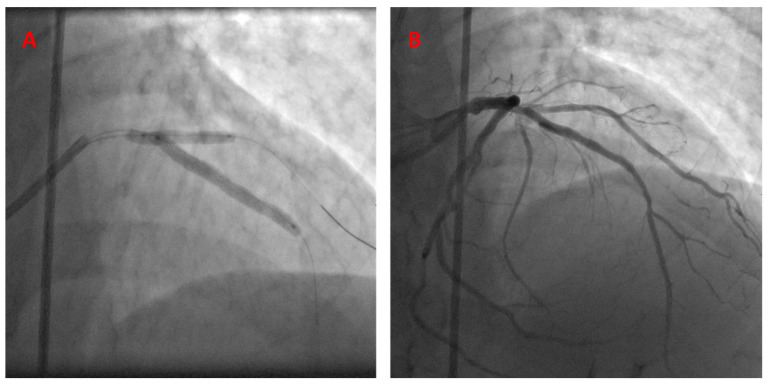
The kissing BS technique, utilized to treat LAD-D1 bifurcation disease (**A**). Post-stent deployment—final angiographic results (**B**).

PCI description: The operator chose a 7 Fr guide catheter for his approach in this case. After engaging the left main coronary artery, 2 wires were advanced antegrade into the LAD and diagonal artery. The LAD lesion was pre-dilated using a 2.75 semi-compliant balloon. A resolute integrity 3.0 × 38 stent was then advanced and positioned in the LAD, starting at the exact takeoff point of the diagonal artery. A semi-compliant 2.75 balloon was then advanced and positioned into the diagonal artery, with the proximal part slightly extending into the LAD. The stent was deployed first, followed by balloon inflation. Angiographic stenosis severity in the LAD was reduced from 70% down to 0%, with a TIMI-3 flow recorded pre- and post-operation. The diagonal artery had mild 30% disease, with a TIMI-3 flow that did not change after intervention (Figure 3, Video S2).

**Figure 4:**
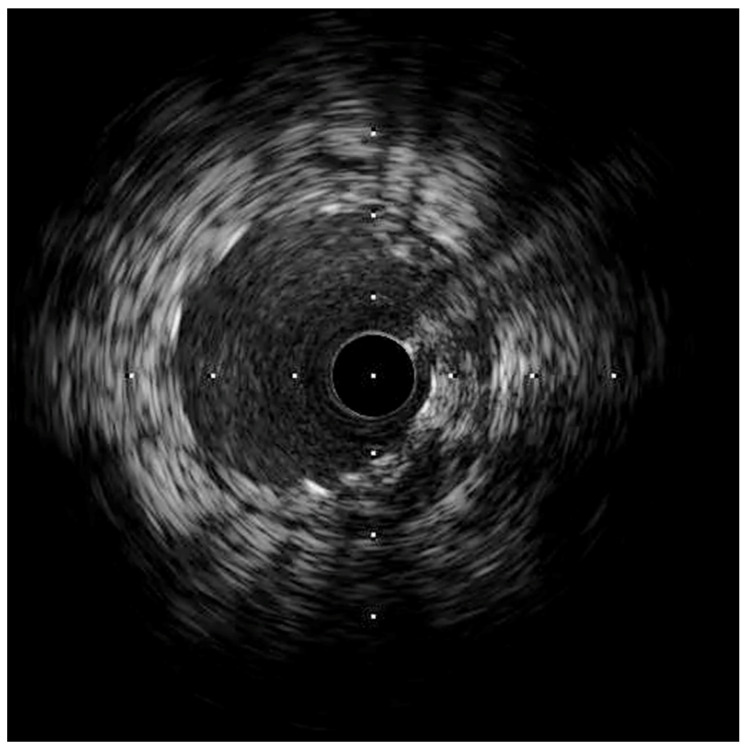
IVUS of the LAD, showing a well-expanded LAD stent with a widely patent diagonal branch ostium.

Following stenting, the intravascular ultrasound (IVUS) showed a well-expanded and well-positioned stent in the LAD, with the preservation of a widely patent diagonal ostium. The ostium of the LAD had atherosclerosis with a minimal luminal area of 5 mm^2^, which was considered not to be significant (Figure 4).

**Figure 5:**
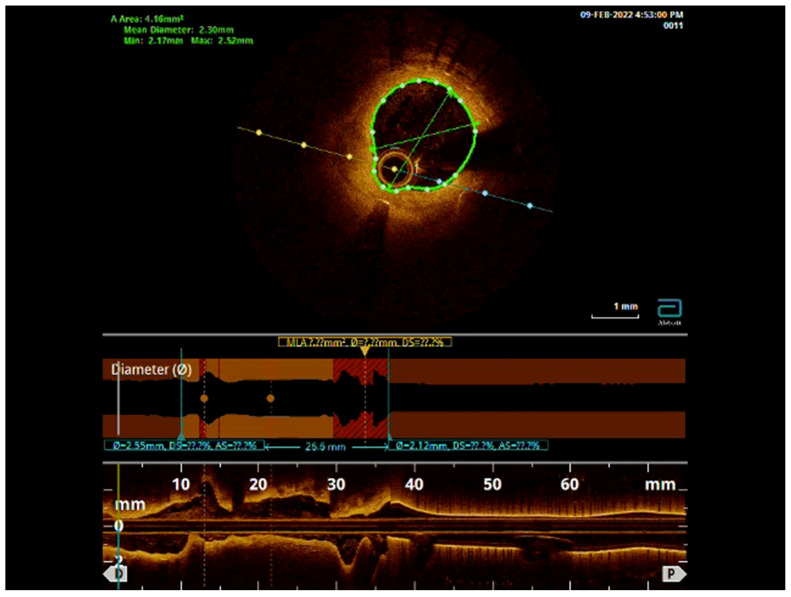
OCT pullback showing the well-positioned LAD stent with mild ISR but with preservation of the diagonal branch ostium.

Follow-up: The patient presented 4 years later with recurrent angina symptoms. A coronary angiogram showed significant progression of coronary artery disease (CAD) in all coronary arteries, including 50% in-stent restenosis of the LAD and 80% stenosis of the ostial diagonal artery, in addition to the progression of disease in the LCx and RCA disease. Optical coherence tomography (OCT) showed a well-positioned stent in the LAD, without any struts covering the origin of the diagonal artery. PCI was then performed for the diagonal artery and the proximal LAD, in addition to other vessels. There was no need to intervene on the mid-LAD. The bifurcation remained preserved (Video S3, Figure 5).

Case # 2

**Figure 6:**
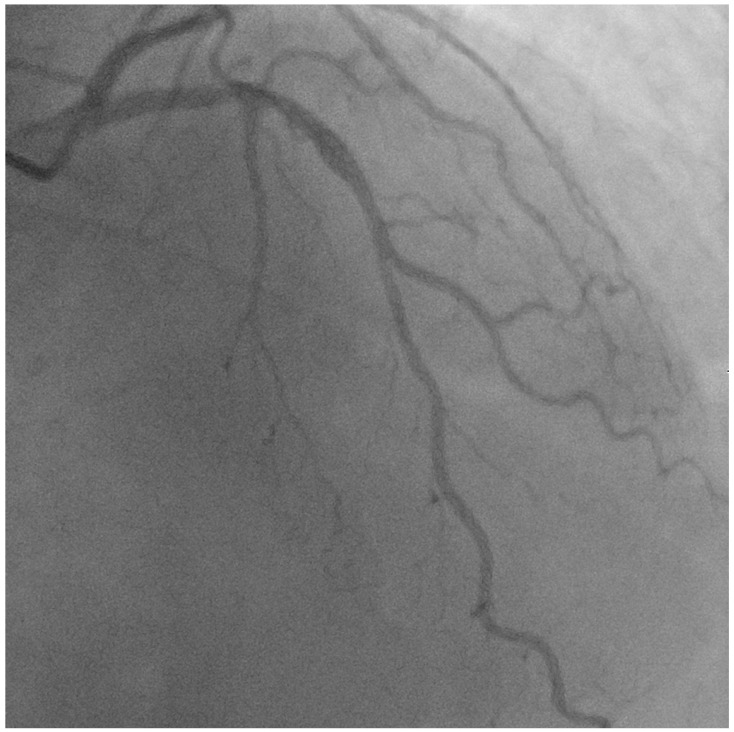
Angiogram showing mid-LAD disease, which was determined to be hemodynamically significant by iFR.

A 50-year-old man with a past medical history of hypertension and hyperlipidemia presented with unstable angina. A coronary CT angiogram showed obstructive disease in the LAD. He was referred for invasive coronary angiography, which showed a 70% lesion in the mid-LAD. A fractional flow reserve (FFR) was performed and showed hemodynamically significant disease at maximal hyperemia; therefore, a PCI was planned (Figure 6, Video S4).

**Figure 7:**
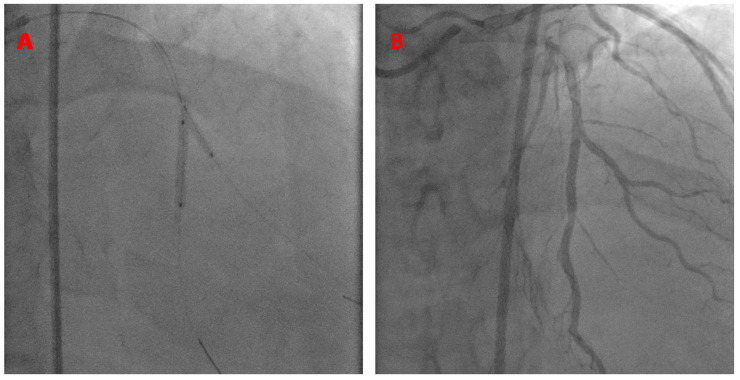
K-BS intervention on a mid-LAD/diagonal bifurcation. The stent is in the mid-LAD and the balloon is in the diagonal branch (**A**). Final angiographic results (**B**).

A 7 French JCL guide catheter was used to engage the left main coronary artery. The kissing BS technique was performed using a Xience Alpine 2.5 × 23 mm stent in the LAD (18 atm) and a 2.5 × 15 mm balloon in the diagonal branch (14 atm), as shown below (Figure 7).

This patient presented 2 years later with similar symptoms of chest pain. He was referred for coronary angiography, which showed the patent LAD stent and native diagonal artery to be without significant disease. The FFR of the LAD was negative. No intervention was made at this time. The patient was seen in follow-ups 4 years post-intervention and continued to progress well clinically, without needing any further interventions (Video S5).

Case # 3

**Figure 8:**
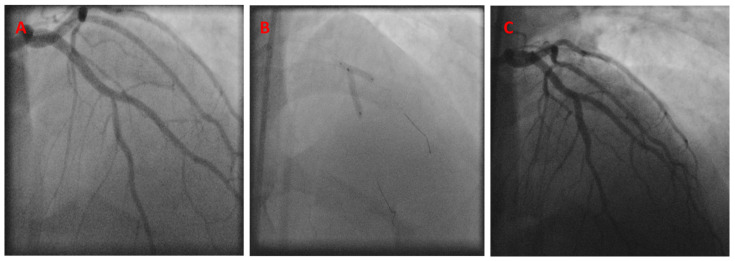
Initial angiogram showing mid-LAD disease (**A**). The K-BS intervention, using a balloon in the diagonal branch and a stent in the LAD (**B**). Final angiographic result (**C**).

A 41-year-old man with hypertension, hyperlipidemia, type II diabetes mellitus, active smoking, a history of stroke, chronic obstructive lung disease, and morbid obesity presented with unstable angina. A diagnostic coronary angiogram showed severe 70–80% stenosis in the mid-LAD, right at the diagonal branch takeoff point. PCI was then performed using an 8 French JCL-4 guide catheter. The kissing BS technique was performed at the LAD/diagonal bifurcation, using a 3.0 × 18 mm Cypher stent in the mid-LAD and a 3.0 × 15 balloon in the diagonal branch, with successful angiographic results (Figure 8).

**Figure 9:**
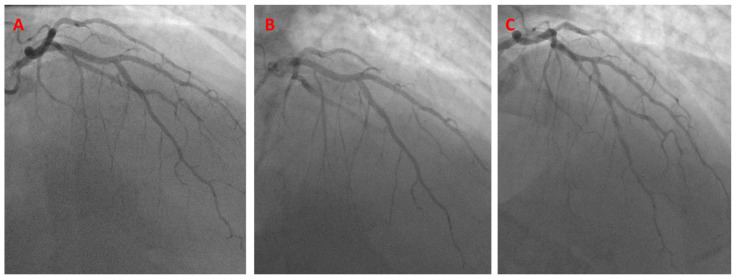
Coronary angiogram recorded 4 years post-PCI, showing a stable LAD/diagonal bifurcation (**A**). Coronary angiogram recorded 6 years post-PCI, showing a stable LAD/diagonal bifurcation (**B**). Coronary angiogram recorded 9 years after the initial PCI, showing the mid-LAD in-stent restenosis (**C**).

The patient presented again 9 years later with in-stent restenosis (ISR) of the mid-LAD and required a repeat PCI using a 2.5 × 28 Xience Aline stent. The patient continued to come back with recurrent ISR over the next follow-up years until he passed away, 11 years after the index procedure. A coronary angiogram performed 11 years later showed a patent diagonal artery without any obstructive ostial disease (Figure 9).

## 6. Brief Summary of the Five Remaining Cases

Case #4: A 61-year-old man with a Medina 0,1,0 lesion. A LAD stent was employed with K-BS in the diagonal branch. The patient returned 9 months later with 90% stenosis in the proximal diagonal artery, which required stenting. He was progressing well clinically 4 years after the index procedure, without repeat coronary interventions being needed.
Case #5: A 55-year-old man with ostial LCx disease, who received K-BS treatment with an LCx stent and balloon in the LAD. The patient presented again 4 years later with the progression of LAD/LCx/LM disease and was referred for coronary artery bypass surgery.
Case #6: A 62-year-old man with unstable angina, who was found to have significant ostial LAD disease. He received a stent in the LAD and a balloon in a Ramus (K-BS). He underwent a follow-up angiogram 2 years later that showed a patent LAD and a Ramus. He continued to progress well clinically 2 years after the index procedure.
Case #7: A 76-year-old patient who underwent a K-BS in the LAD/D1 bifurcation lesion. No follow-up angiogram was required, and he continued to progress well clinically 4 years after the index procedure.
Case #8: A 67-year-old patient with severe peripheral arterial disease. The patient underwent K-BS in an ostial LAD (stent)/Ramus (balloon) bifurcation lesion. A follow-up angiogram 4 months later showed patent anatomy. The patient continued to progress well 1 year after coronary intervention.

## 7. Discussion

Medina 0,1,0 and 0,0,1 bifurcation lesions, although simple, can present significant technical difficulty during percutaneous coronary interventions. There has not been a consensus regarding the optimal management of those lesions, particularly in the case of 0,0,1 lesions, which have been shown in multiple cohorts to carry a worse prognosis compared to more complex bifurcations [7,9]. Several management strategies have been investigated, including plain balloon angioplasty, drug-coated balloons, and stenting, including single-stent and two-stent strategies. Drug-coated balloons have emerged as a promising technique, especially for 0,0,1 lesions [10,11]. Vaquerizo et al. evaluated 49 patients with Medina 0,0,1 lesions and evidence of myocardial ischemia. Those lesions were treated with paclitaxel drug-coated balloons with a 45-second inflation. Bail-out stenting was required in 14% of the lesions, and, at the 1-year follow-up, 14% of the lesions required repeat revascularization [12]. The single versus two-stent technique has also been investigated, with similar clinical outcomes. Choi et al. evaluated the one- vs. two-stent strategy for Medina 0,0,1 and 0,1,0 lesions, with the rates of MACE at the 800-day follow-up being similar at 14.3% and 13.9%, respectively [6]. Other bifurcation techniques have been described in this space to address these challenging lesions. The flower-petal technique was described initially in 2013 and has been demonstrated to have very favorable short-term results at 9 months, yet its implementation in clinical practice has been limited [13,14]. During the provisional stenting of these lesions, several issues may arise. The major challenge is ensuring adequate coverage of the vessel ostium and the plaque with stent struts. Main-vessel stenting may induce significant worsening at the level of the jailed side branch. This phenomenon has been demonstrated to be mainly related to carina shift [15,16]. Plaque shift can also occur in the non-diseased branch, hence compromising its integrity [16]. Additionally, stent protrusion into the healthy branch when trying to perfectly position the stent exactly at the origin point of the bifurcation could potentially compromise its integrity and make any future intervention attempts challenging. 

In this case series, we describe the outcomes of the kissing balloon-stent technique in eight patients who received long-term follow-ups (Table 1). The duration of follow-ups in our cohort is variable depending on the clinical presentation, ranging from 9 months to 11 years, with an average duration of around 4 years. Seven out of the eight patients underwent follow-up angiograms. There were no immediate post-procedural complications in any of the treated patients. The major follow-up outcome of this technique would be looking into the safety of inflating a balloon in an apparently healthy branch with a ratio of 0.9:1 for balloon-to-vessel diameter, evaluating the rates of side branch stenosis and the rates of re-stenosis of the stented vessel. Three out of the eight cases showed disease progression in the branch where the balloon was inflated (Table 2). Of note, two of those cases (case 1 and case 5) had exhibited some mild non-obstructive disease in the ballooned branch from the beginning. Inflating a balloon in a vessel with plaque formation always carries the risk of plaque disruption or disease acceleration in the ballooned area. Hence, if this technique is to be applied when the ballooned vessel has some minor disease, we recommend that the balloon is either potentially undersized even more or that an alternative bifurcation technique is utilized. Although side-branch dissections were not observed in the current cohort, they do pose a potential complication if a balloon is inflated in an angiographically healthy-appearing branch that is actually diseased. Covering dissections with long stents and the potential conversion to a two-stent strategy would be necessary at that point [17]. Alternatively, when there is a residual intermediate lesion in the side branch post-balloon inflation, the use of a coronary physiological assessment with FFR or iFR could be utilized and has been previously described in the literature along with a provisional stenting approach [18].

The current case series shows that 3 out of the 8 patients had ISR within the stented branches (Table 2). This could potentially be related to stent under-expansion, as the operator may have been hesitant to post-dilate the deployed stent or to dilate it at higher pressures in order not to compromise the side branch. Acute or subacute stent thrombosis was not observed with this technique, although it can potentially happen, especially if the stent in the stented vessel is under-expanded or if there is a side-branch dissection extending into the main vessel. 

The use of intravascular imaging is increasingly being utilized to optimize percutaneous coronary interventions and has been consistently shown to improve outcomes, especially in complex lesions [19,20]. Interestingly, with the increased use of intracoronary imaging, operators probably frequently re-classify the Medina classification of bifurcation lesions. A significant amount of angiographic Medina 0,0,1 and 0,1,0 lesions are probably more complex lesions, which may partially explain the worse clinical outcomes reported with these lesions. The use of intracoronary imaging could be very helpful when assessing candidacy for this technique. An operator can potentially evaluate the integrity of the ballooned branch first and then size the balloon accordingly. Additionally, an operator can use intracoronary imaging to assess the patient for any evidence of plaque disruption after ballooning and, potentially, convert the technique to a two-stent strategy. Imaging the stented vessel can also help to evaluate if the stent has expanded and if post-dilatation is necessary with repeating kissing balloons. Hence, the perfect candidate for this technique would be a patient who has no significant disease in the balloon branch, has a narrow angle between the branches, and who has a soft plaque in the stented branch, where stent under-expansion would be minimized. Table 3 provides a brief summary of the current bifurcation techniques that could be used with Medina 0,1,0 and 0,0,1 bifurcation lesions, along with their pros and cons. 

## 8. Study Limitations

This is a small case series describing the application of the kissing balloon-stent technique in Medina 0,0,1 and 0,1,0 lesions. Due to the small number of patients, the results should be viewed as exploratory and could not be generalized for wider cohorts of patients or compared to other bifurcation techniques. No demographic conclusions could be drawn either, as the majority of the patients were white males. Operator experience in bifurcation stenting is also necessary for this technique, in case conversion to a two-stent approach is required. Further and larger studies with more systematic follow-up procedures are needed for better validation and to enable comparison with more widely described bifurcation techniques.

## 9. Conclusions

The kissing balloon-stent technique may be useful for addressing simple Medina 0,0,1 or 0,1,0 bifurcation lesions. One should exercise caution and try to avoid this technique if there is any evidence of plaque formation in the balloon branch. Alternatively, the use of intracoronary imaging is encouraged, to size the balloon accordingly or to assess for any plaque disruption in the ballooned segment. Further and larger studies are needed for more validation of this technique and for comparison with more conventional bifurcation PCI practices.

## Figures and Tables

**Table 1:** Summary of 8 cases in which the kissing balloon-stent technique was utilized.

Patient	Age	Lesion	Intervention	Follow-Up Angiogram	Clinical Follow-Up
Case 1	56 yo	LAD/D1	LAD stent, D1 balloon	4 years	4 y, SR
Case 2	50 yo	LAD/D1	LAD stent, D1 balloon	2 years	4 y, NR
Case 3	41 yo	LAD/D1	LAD stent, D1 balloon	11 years	11 y, NR
Case 4	61 yo	LAD/D1	LAD stent, D1 balloon	9 months	9 m, SR
Case 5	55 yo	Ostial LCx/ LAD	LCx stent/LAD balloon	4 years	4 y, SR
Case 6	62 yo	Ostial LAD/Ramus	LAD stent/Ramus Balloon	2 years	2 y, NR
Case 7	76 yo	LAD/ D1	LAD balloon/D1 stent	None	4 y, NR
Case 8	67 yo	Ostial LAD/Ramus	LAD stent/Ramus Balloon	4 months	1 y, NR

SR: symptom recurrence, NR: no symptom recurrence.

**Table 2:** The lesion outcomes of the 8 cases.

Patient	Stented Lesion	Ballooned Lesion
Case 1	LAD ISR	D1 is an 80% lesion
Case 2	LAD patent	D1 is patent
Case 3	LAD ISR	D1 is patent
Case 4	LAD patent	D1 is a 90% lesion
Case 5	LCx disease progression	LAD disease progression
Case 6	LAD is patent	Ramus is patent
Case 8	LAD is patent	Ramus is patent

ISR: In-stent restenosis.

**Table 3:** Bifurcation techniques summary for Medina 0,0,1 and 0,1,0 lesions.

Technique	Pros	Cons
Single-stent strategy		
Provisional crossing side branch	Single stent Simple technique Can easily convert to a two-stent technique	Side branch occlusion/jailing
Provisional Nailing of the ostium	Single stent Simple technique	Missing the ostium/carina or plaque shift into the side branch
Kissing BS	Single stent Minimizes plaque and carina shift	Plaque disruption–dissection of balloon branch
Two-stent strategy		
Culotte	Used in bifurcation angles of <70 degrees	Lose main vessel wire access Can be difficult to re-cross
T stenting	Used for 90-degree angles	Can miss the ostium
DK Crush	Maintain main vessel wire access Superiority of data with LM disease	Technically challenging/multiple steps Longer time. Can be difficult to re-cross
Single-stent conversion to a two-stent (bailout) procedure		
Reverse culotte	Reconstructing bifurcation	Difficulty re-crossing
Reverse T stenting	Simple technique	Too much protrusion into the main branch

## Data Availability

No extra data is available anywhere else.

## Supplementary Materials

The following supporting information can be downloaded at: https://www.mdpi.com/article/10.3390/jcm13092645/s1, Video S1: Patient 1 angiography in RAO-Cranial view depicting mid LAD-D1 Medina 0,1,0 lesion. Video S2: Patient 1 angiography in RAO-Cranial view depicting mid LAD-D1 lesion post percutaneous coronary intervention using kissing balloon-stent technique. Video S3: Optical coherence tomography pullback showing the well-positioned LAD stent with mild ISR but with preservation of the diagonal branch ostium. Video S4: Patient 2 angiography in AP-Cranial view showing mid LAD-Diagonal bifurcation medina 0,1,0 lesion. Video S5: Patient 2 angiography in AP-Crania view depicting mid LAD-Diagonal bifurcation lesion post percutaneous coronary intervention using kissing balloon-stent technique.
